# Effects of common eye diseases in children and their treatment measures on ocular surface homeostasis: A review

**DOI:** 10.1097/MD.0000000000038784

**Published:** 2024-07-12

**Authors:** Zongyue Lv, Zhengyang Tao, Jing He, Jiao Wang, Zhihong Lin, Zefeng Kang, Hongwei Deng

**Affiliations:** a The Second Clinical Medical College, Jinan University, Shenzhen, China; b Shenzhen Eye Hospital, Jinan University, Shenzhen, China; c Affiliated Shenzhen Maternity & Child Healthcare Hospital, Southern Medical University, Shenzhen, China; d Department of Ophthalmology, Ophthalmic Hospital, Chinese Academy of Traditional Chinese Medicine, Beijing, China.

**Keywords:** Allergic conjunctivitis, ametropia, dry eye disease, homeostasis, ocular surface, pediatric, strabismus

## Abstract

Ocular surface homeostasis plays a vital role in maintaining of eye health. Dry eye disease is one of the prominent and typical manifestations of disruption of ocular surface homeostasis that leads to the worsening of ocular surface homeostasis that leads to the worsening of ocular surface disease when it interacts with other pathogenic factors. However, disruption in ocular surface homeostasis in children is often overlooked because of the current methods of assessing ocular surface homeostasis. This review summarizes the main factors affecting ocular surface homeostasis in children, with the aim of drawing the attention of clinicians to the disruption of ocular surface homeostasis in children when dealing with such diseases. Ocular surface homeostasis involves several interrelated components, each of which plays a nonnegligible role in ocular surface homeostasis. Unlike adults, children have a stronger lacrimal gland secretion capacity and milder symptoms when there is a slight disruption of the ocular surface homeostasis. In addition, children’s expressive abilities were weaker. Therefore, dry eye in children is often ignored by doctors and parents, and clinicians should pay more attention to the protection of ocular surface homeostasis when treating children with these diseases. Therefore, there is a need for diagnostic criteria for dry eye disease specific to children.

## 1. Introduction

Ocular surface homeostasis is jointly maintained by the ocular surface tissues and through the tear secretion mechanism, which are interrelated to guide the production of the tear film. Zhang et al proposed the concept of the ocular surface microenvironment, which mainly includes ocular surface tissues, immune cells, stromal cells, small molecules, and microorganisms. Changes in any of these biological entities may lead to damage to ocular surface homeostasis and affect tear film stability, which in turn leads to the development of dry eye disease.^[1]^ In 2017, the New International Consensus on Dry Eye Disease defined dry eye disease as a multifactorial ocular surface ailment characterized by an unbalanced tear film accompanied by ocular symptoms.^[2]^ The factors affecting ocular homeostasis in children differ slightly from those in adults. For example, sex hormones do not significantly influence ocular homeostasis in children. Likewise, symptoms are mild in children with minor damage to the ocular surface damage. In such a situation, the strong secretion capacity of the lacrimal glands leads to negligible damage to ocular surface homeostasis in children. In this case, the strong secretory capacity of the lacrimal gland leads to milder symptoms in children who suffer disruption of ocular surface homeostasis.^[3]^ A research report on 3433 healthy Japanese high school students showed that the proportion diagnosed with dry eye disease was 12.4%, with 43.4% of the students reporting severe dry eye symptoms.^[4]^ Dry eye disease is thought to affect only older adults; however, with the popularization of computers, mobile phones, and online courses, the incidence of dry eye disease is dramatically increasing in children.^[5,6]^ Thus, the occurrence of damage to the ocular surface homeostasis in children requires more attention.

## 2. Effect of allergic conjunctivitis and their treatment measures on ocular surface homeostasis

### 2.1. Effect of allergic conjunctivitis on ocular surface homeostasis

Allergic conjunctivitis mainly affects goblet cell function and damages the cornea and conjunctiva, leading to an imbalance in ocular surface homeostasis. Allergic conjunctivitis is 1 of the most common diseases in pediatric ophthalmology. Recent studies have shown that nearly 30% of students in Shanghai suffer from allergic conjunctivitis, with a higher prevalence among young children.^[7,8]^ Allergic conjunctivitis is divided into acute and chronic allergic conjunctivitis. Acute allergic conjunctivitis is caused by IGe-mediated mast cell degranulation, and its main symptoms include itchiness, lacrimation, and transient symptoms of persistent swelling. Chronic allergic conjunctivitis is associated with the sustained activation of mast cells and cytokines produced by eosinophils and Th2 cells and is primarily characterized by the loss of vision and the occurrence of pain.^[9]^ Seasonal allergic conjunctivitis and perennial allergic conjunctivitis are the most common forms of allergic conjunctivitis in children. The main symptoms in children are itching of the eye, conjunctival congestion, edema, nipples, follicles, and other signs.^[10,11]^

The tear film is composed of 3 layers: mucin, oil (lipid), and water (aqueous). The mucin layer helps adhere the tear film to the eye surface and maintains the stability of the precorneal tear film. MUC5AC is the most important mucin in the mucin layer, which captures pollen and other eye surface allergens, removes them from the eye surface, and transports them to the lacrimal excretory system to protect the ocular surface. The synthesis and secretion of MUC5AC are strictly controlled by goblet cells. Recent studies have shown that allergic inflammation affects the goblet cell status.^[12,13]^ Garcia-Posadas et al reported that allergic conjunctivitis can cause type I allergic reactions. Allergic reactions cause the release of various types of cytokines by inflammatory cells, whereas activation of Th2 cytokines increases the number of goblet cells and IL-13 significantly stimulates the proliferation of goblet cells.^[14,15]^ An increase or decrease in mucin levels can lead to a decrease in tear film stability, which in turn can lead to dry eye syndrome. An animal study by Toda et al demonstrated that goblet cells exhibit hypersecretion during the acute phase of allergic conjunctivitis, followed by degeneration or damage of goblet cells.^[16]^ Both atopic keratitis and vernal keratoconjunctivitis result in damage to the conjunctiva and cornea, disrupting ocular surface homeostasis. Hu et al recruited 12 patients with atopic keratoconjunctivitis and 6 patients with vernal keratoconjunctivitis and found that all patients had a higher level of squamous metaplasia of the conjunctival epithelium and lower corneal sensitivity than healthy controls.^[17]^ Metaplasia of the conjunctiva impairs the epithelial barrier of the ocular surface, which causes increased abrasion stress and epithelial damage, and decreases the proliferation of goblet cells.^[17–19]^ Decreased corneal sensitivity indicates damage to the corneal epithelium or stroma layer,^[20]^ both of which lead to an unstable tear film and disturbance of ocular surface homeostasis.

### 2.2. Effects of eye rubbing on ocular surface homeostasis

In children with allergic conjunctivitis, conjunctival mast cells are the key factor causing itching in the eyes. The limited expression ability of children and undetectable early symptoms after the use of dual agents often contribute to a missed diagnosis. Frequent eye rubbing by children not only alleviates the unpleasant feelings caused by allergic conjunctivitis^[21]^ but can also damage the corneal epithelium, which may lead to worsening inflammation on the ocular surface.^[22,23]^ As a result, the cycle of itch-eye rubbing-itch might be triggered, and the child may rub the eye more frequently, which may lead to remodeling of corneal tissue, especially keratoconus.^[24,25]^ The formation of keratoconus combined with frequent eye rubbing causes the release of cytokines by corneal epithelial cells, which leads to strong inflammatory responses that induce apoptosis of corneal stromal cells and fibroblasts. This inflammatory cycle ultimately leads to disruption of ocular surface homeostasis.^[26]^ In addition, constant stress on the meibomian gland tissue caused by eye rubbing can distort the meibomian gland, which may result in dysfunction of the meibomian gland and dry eye.^[27,28]^

### 2.3. The interaction of allergic conjunctivitis and dry eye further disrupts the ocular surface homeostasis

Recent evidence has shown that dry eye in children is associated with allergic ocular surface diseases.^[29]^ Most children with allergic conjunctivitis also suffer from dry eye.^[23]^ There is no doubt about the relationship between dry eye and allergic eye surfaces in children, and these 2 diseases share certain clinical symptoms. Regardless of the initial event, dry eye persists due to the vicious cycle of ocular surface inflammation, instability, and hyperosmolarity of the tear film.^[30,31]^ In contrast, the loss of ocular mucin caused by dry eye disease leads to failure of timely clearance of allergens from the ocular surface, which further leads to the aggravation of allergic ocular surface diseases. During treatment, negligence of 1 of these diseases may accelerate eye injury and worsen prognosis, emphasizing that clinicians should carefully investigate the coexistence of dry eye and allergic ocular surface diseases in children.^[18]^

### 2.4. Effect of drugs on allergic conjunctivitis on ocular surface homeostasis

Antihistamines and mast cell stabilizers are widely used as first-line drugs for the treatment of allergic conjunctivitis in children.^[32]^ A recent study found a small number of patients with dry eye after the use of dual agents.^[33]^ Villareal et al reported that the volume of tears in mice significantly decreased 15 and 45 minutes after the administration of olopatadine.^[34]^

## 3. Effect of ametropia and its treatment measures on ocular surface homeostasis

### 3.1. Influence of ametropia on ocular surface homeostasis

Ametropia is a common eye disease in children and China has the highest incidence of myopia among young people in Asia. With the global COVID-19 pandemic, the increase in online courses has led to a marked increase in children’s electronic screen time, which has aggravated ametropia. Wang et al used 5M ophthalmometers, slit-lamp, and questionnaire to evaluate the symptoms of dry eye in myopic adolescents and found that the lipid secretion of meibomian glands in myopic adolescents increased abnormally, which may cause dysfunction of the meibomian gland.^[35]^ Another reason for disruption of ocular surface homeostasis caused by ametropia may be that ametropia often leads to increased blinking and visual fatigue in children. Ineffective and frequent blinking can lead to decreased tear film stability, thus disrupting ocular surface homeostasis.^[36,37]^ Furthermore, the extended use of electronic screens may disturb homeostasis of the eye surface. Cremers et al found that the extended use of electronic screens can reduce blink frequency and increase incomplete blink frequency, which may lead to rapid evaporation of tears, instability of the tear film, and increase in inflammatory factors on the ocular surface.^[38]^ Moreover, tear production is lower at night, and screen use is more likely to cause dry eye symptoms.^[39]^ In a study by Uchino et al, the concentration of MUC5AC in tears was significantly decreased in patients with dry eyes who used electronic screens, which was also 1 of the reasons for the damage to ocular surface homeostasis.^[40]^

### 3.2. Influence of orthokeratology on ocular surface homeostasis

Currently, orthokeratology (OK) is widely used to control myopia in teenagers. According to the literature, many children wearing OK lenses may suffer from dry eye, which may be caused by the influence of wearing OK lenses on the goblet cells and the stability of the tear film. Carracedo et al found no significant difference in the goblet cell count after 1 month of wearing OK lenses.^[41]^ However, a study by Yang et al found that the tear film breakup time decreased significantly from 6 months to 12 months after wearing OK lenses, which may be related to the changes in tear mucin, corneal epithelium, and meibomian gland function. Moreover, the patient also developed atrophy of the meibomian glands 12 months after wearing the OK lenses.^[42]^

Cho et al found that there were significant differences in the distribution of the first tear film breakup time between children wearing OK lenses to control myopia and healthy controls (*P* = .03), which might be related to the reduction in the smoothness of the corneal surface by the OK lenses. High-order aberrations (HOAs), representing ocular surface irregularities caused by the anterior corneal surface and tear film, were also higher in children wearing OK lenses than in healthy children (*P* < .0001).^[43]^ Our team conducted a meta-analysis related to the use of OK lenses before October 19, 2020, and found that they can affect the stability of the tear film but have no significant effect on tear secretion.^[44]^ A number of factors can influence the results of this study, such as the measurement method used to measure the breakup time of the tear film, the preparation for wearing OK lenses, night sleep time, and the duration of measures. Therefore, studies on the effect of orthokeratography on ocular surface homeostasis require stricter control of these variables.

## 4. Effects of strabismus and its treatment measures on ocular surface homeostasis

### 4.1. Effects of strabismus on ocular surface homeostasis in children

Strabismus is caused by an imbalance in extraocular muscle strength in both eyes.^[45]^ Giannaccare et al proposed that deviation from eye position due to strabismus may lead to increased exposure of the conjunctival area, which increases the risk of conjunctival damage and thus may lead to instability of the tear film. Inflammatory events may be activated by both conjunctival injury and the hypertonic state of tears caused by the unstable tear film, which may further damage ocular surface homeostasis.^[46]^ Gao et al found that even children with strabismus did not develop dry eye symptoms, whereas the levels of IL-6 and TNF-α increased significantly in their tears.^[47]^

### 4.2. Effect of Botox for strabismus on ocular surface homeostasis

The injection of botulinum toxin, mainly type A, has become a common treatment for children and adults with strabismus.^[48]^ According to reports, 63% of patients experience weakened function of the orbicularis oculi muscle after receiving botulinum toxin injections, leading to a decrease in blink frequency and resulting in the occurrence of dry eye symptoms.^[49]^ Sekeroglu et al found that botulinum toxin injection may also spread to the lacrimal gland, thus affecting its secretion and leading to damage to ocular surface homeostasis.^[50]^

### 4.3. Effect of correction surgery on ocular surface homeostasis

Correction surgery is a common treatment for strabismus, and some patients may develop dry eye after surgery.^[51]^ Li et al found that the cause of postoperative dry eye may be related to the surgical incision location. The main incisions in strabismus surgery include limbal and fornix incisions, where the former may lead to denervation of the cornea and affect ocular surface homeostasis.^[52]^

## 5. Effects of malnutrition on ocular surface homeostasis

**Figure 1.**
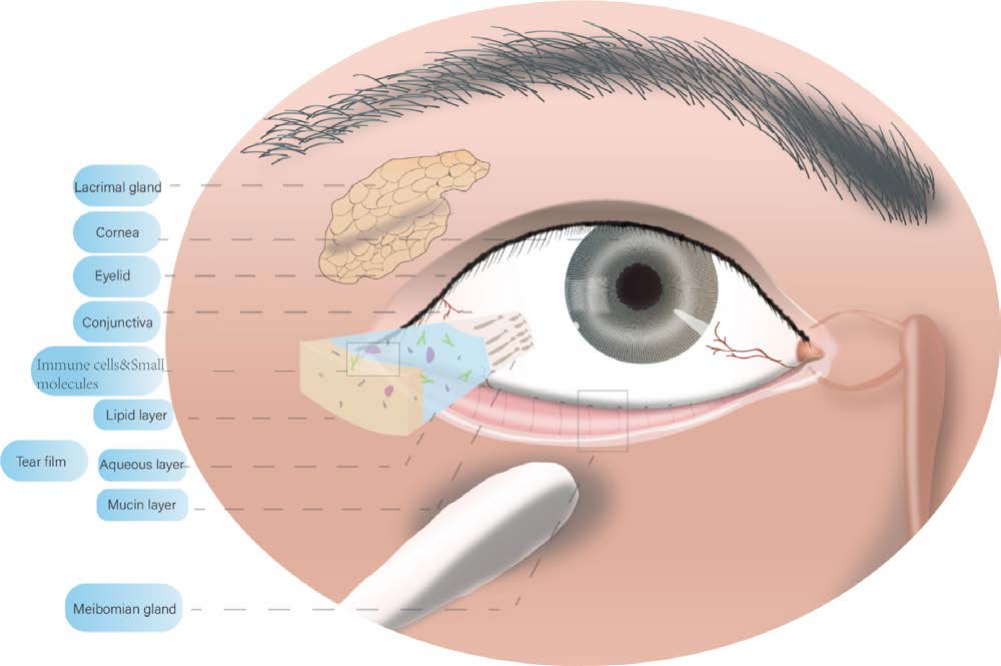
Vulnerable components of the pediatric ocular surface microenvironment.

Ocular surface homeostasis is maintained by various factors (Fig. 1). In addition to the common eye diseases described in this review, some common systemic diseases in children may affect ocular surface homeostasis. According to recent studies, one-third of the children in countries with a gross domestic product of less than $15,000 are vitamin A deficient.^[53]^ Vitamin A is associated with the normal development and growth of organs as well as the establishment of immunity and tissue differentiation. Vitamin A deficiency (VAD) is a leading cause of preventable blindness in young children.^[54,55]^ During progressive VAD, a number of changes occur in the eye, including squamous metaplasia of the conjunctival epithelium, corneal dryness, ulceration, and necrosis, which may cause dry eye disease. Children with dry eye disease have decreased MUC5AC levels and reduced ability to trap and remove allergens, which is a high-risk factor for allergic conjunctivitis. Once allergic conjunctivitis occurs and cytokines are activated, the demand for vitamin A is significantly increased, which aggravates VAD to form a vicious cycle of vitamin A deficiency-dry eye allergic conjunctivitis.^[56–58]^

## 6. Summary and prospect

This article reviews common eye diseases in children and the mechanisms by which these treatments disrupt ocular surface homeostasis. Ocular surface homeostasis involves several interrelated components, each of which plays a nonnegligible role in ocular surface homeostasis. Unlike adults, children have a stronger lacrimal gland secretion capacity and milder symptoms when there is slight disruption of ocular surface homeostasis. In addition, children’s expressive abilities were weaker. Therefore, dry eye in children is often ignored by doctors and parents, and clinicians should pay more attention to protecting ocular surface homeostasis when treating children with these diseases. The authors believe that, at the clinical level, there is a need for diagnostic criteria for dry eye disease that are specific to children. In future studies on dry eye disease in children, more attention should be paid to the control of variables such as tear sampling time and children’s sleep time to obtain more reliable study data.

## Acknowledgments

The authors have no relevant financial or nonfinancial interests to disclose. This work was financially supported by the national key R&D plan “Intergovernmental International Scientific and Technological Innovation Cooperation” (2022YFE0132600), the Sustainable development project of Shenzhen Science and Technology Commission (KCXFZ20211020163814021), and the role of ALDH3A1 gene in the process of scleral remodeling in myopic mice (JCYJ20230807114604008).

## Author contributions

**Data curation:** Zongyue Lv, Zhengyang Tao, Jing He.

**Methodology:** Zongyue Lv, Jing He, Zhihong Lin, Zefeng Kang, Hongwei Deng.

**Writing – original draft:** Zongyue Lv.

**Formal analysis:** Zhengyang Tao, Jiao Wang.

**Conceptualization:** Jiao Wang, Hongwei Deng.

**Investigation:** Zhihong Lin.

**Writing – review & editing:** Zefeng Kang, Hongwei Deng.
